# Efficacy of Nucleot(s)ide Analogs Therapy in Patients with Unresectable HBV-Related Hepatocellular Carcinoma: A Systematic Review and Meta-Analysis

**DOI:** 10.1155/2017/7075935

**Published:** 2017-03-15

**Authors:** Lingling He, Xiaoli Liu, Yalin Zhao, Shuan Zhang, Yuyong Jiang, Xianbo Wang, Zhiyun Yang

**Affiliations:** ^1^Center of Integrative Medicine, Beijing Ditan Hospital, Capital Medical University, Beijing 100015, China; ^2^Collaborative Innovation Center of Infectious Diseases, Capital Medical University, Chaoyang, Beijing 10015, China

## Abstract

*Aim*. To determine whether nucleot(s)ide analogs therapy has survival benefit for patients with HBV-related HCC after unresectable treatment.* Method*. A systematic search was conducted through seven electronic databases including PubMed, OVID, EMBASE, Cochrane Databases, Elsevier, Wiley Online Library, and BMJ Best Practice. All studies comparing NA combined with unresectable treatment versus unresectable treatment alone were considered for inclusion. The primary outcome was the overall survival (OS) after unresectable treatment for patients with HBV-related HCC. The secondary outcome was the progression-free survival (PFS). Results were expressed as hazard ratio (HR) for survival with 95% confidence intervals.* Results*. We included six studies with 994 patients: 409 patients in nucleot(s)ide analogs therapy group and 585 patients without antiviral therapy in control group. There were significant improvements for the overall survival (HR = 0.57; 95% CI = 0.47–0.70;* p* < 0.001) and progression-free survival (HR = 0.84; 95% CI = 0.71–0.99;* p* = 0.034) in the NA-treated group compared with the control group. Funnel plot showed that there was no significant publication bias in these studies. When it comes to antiviral drugs and operation method, it also showed benefit in NA-treated group. At the same time, overall mortality as well as mortality secondary to liver failure in NA-treated group was obviously lesser. Sensitivity analyses confirmed the robustness of the results.* Conclusions*. Nucleot(s)ide analogs therapy after unresectable treatment has potential beneficial effects in terms of overall survival and progression-free survival. NA therapy should be considered in clinical practice.

## 1. Introduction

Hepatocellular carcinoma (HCC) is one of the most common cancers all over the world, especially in developing countries. In 2012, about 745,500 people died of liver cancer in the world, of which China alone accounted for about 50% [1]. The unequal distribution of HCC between developing and developed countries suggests that a variety of environmental factors contribute to the development of this cancer. In most developing countries including China, chronic hepatitis B virus (HBV) infection accounts for the majority of primary liver cancer [2]. Antiviral therapy is the main preventive strategy for HCC with chronic HBV infection [3]. Surgical resection is a curative treatment option for patients with small, solitary nodules without underlying cirrhosis [4]. Antiviral therapy is believed to improve survival rate in patients with HBV-infected HCC (HBV-HCC) [5, 6]. However, the efficacy of antiviral therapy with nucleot(s)ide analogs (NA) for unresectable HBV-HCC is not well described.

Numerous studies to date have evaluated the effects of NA therapy in patients with unresectable HBV-HCC. However, as a result of differences in sample sizes, accuracies of the statistical data, study populations, and interventions, the results remain inconclusive, and evidence-based confirmation by large-scale clinical trials is still lacking. In this study, a meta-analysis was performed according to the Cochrane Handbook [7]. We analyzed the data using hazard ratios (HR), which are most appropriate for time-to-event outcomes.

## 2. Methods

### 2.1. Searching Strategy and Selection of Trials

We performed a systematic search on 6 December 2016. Twelve electronic databases were searched including PubMed, OVID, EMBASE, Cochrane Databases, Elsevier, Wiley Online Library, and BMJ Best Practice. MeSH terms combined with free text words including “hepatitis B,” “liver neoplasm,” “liver cancer,” “hepatocellular carcinoma,” “radiofrequency ablation,” “microwave ablation,” “cryoablation,” “percutaneous ethanol injection,” “chemoembolization,” “lamivudine,” “adefovir,” “telbivudine,” “entecavir,” “tenofovir,” “clevudine,” “nucleot(s)ide analogues,” and “antiviral” were searched. A manual search of the reference lists of all included studies and relevant reviews was performed.

### 2.2. Study Selection and Exclusion Criteria

Inclusion criteria of the selected studies were as follows: (1) study design: cohort studies and randomized controlled trials were considered; (2) patient population: adult patients with HBV-HCC were included; (3) therapy for HCC: unresectable treatment including radiofrequency ablation, microwave ablation, cryoablation, and chemoembolization was considered; (4) antiviral treatment: it consisted in combined nucleot(s)ide analogs with unresectable treatment as therapy group compared with unresectable treatment without antiviral treatment as control group; (5) survival was analyzed in the study; relationships between treatment of nucleot(s)ide analogs and prognostic indicators such as recurrence-free survival (RFS), progression-free survival (PFS), and overall survival (OS) were evaluated; (6) hazard ratio (HR), odds ratio (OR), relative risk (RR), and 95% confidence intervals (CI) that could be obtained directly from the full article or indirectly calculated with relevant software based on the data provided in the graphics and tables were expressed; (7) only the newest studies or the ones with higher quality were retained if the data were repeated in different studies; (8) studies in English were included.

Exclusion criteria were as follows: (1) cell or animal studies, case reports, letters, reviews, and meta-analyses and (2) studies including patients coinfected with HIV or HCV were excluded.

### 2.3. Assessment of Study Quality

Jadad standard was used to assess the quality of included randomized controlled trials (RCT) study [8]. Newcastle-Ottawa Scale was used to assess the quality of the cohort studies.

### 2.4. Data Extraction and Validity Assessment

Two independent investigators extracted data using a predefined form, including general information, baseline characteristics of patients, antiviral methods, unresectable treatment methods, and outcomes from each study. All of the relevant texts, tables, and figures were reviewed for data extraction. Discrepancies in the information obtained by these two investigators were resolved by discussion among all the authors.

### 2.5. Statistical Analysis

This meta-analysis was reported according to the preferred reporting items for systematic reviews and meta-analyses (PRISMA) statement [9]. It was performed in accordance with the recommendations of Cochrane Handbook. HR was applied as a summary statistic for time-to-event outcomes like OS and PFS. HR and its 95% confidence intervals (95% CI) of each study were calculated by a method described by Tierney et al. and Zou et al. [10, 11]. The overall HR < 1 favored the NA-treated group. In the subgroups, risk ratio (RR) and the corresponding 95% CI were used to compare the incidence of mortality between the NA-treated group and control group. RR < 1 represented a lower rate of mortality of the NA-treated group.

RevMan 5.2 (Cochrane Collaboration, Oxford, UK) and Stata for Windows version 11.0 (StataCorp, College Station, Texas, USA) were used for data analysis [12]. Statistical heterogeneity was assessed with* I*^2^ and *χ*^2^ guided by the Cochrane Handbook. According to Higgins et al. and Gan et al. [7, 13],* I*^2^ < 25%, 25% <* I*^2^ < 50%, and* I*^2^ > 50% were considered as low, moderate, and high amounts of heterogeneity, respectively. A fixed-effect model was applied if heterogeneity was not substantial (*I*^2^ < 25%). On the other way round, a random-effect model was applied.

Publication biases were evaluated by funnel plot. Sensitivity analyses were used to evaluate the reliability of the results. In the sensitivity analysis, exclusion of single study at one time was assessed to investigate its influence of individual study.

## 3. Results

### 3.1. Characteristics of the Studies

**Figure 1:**
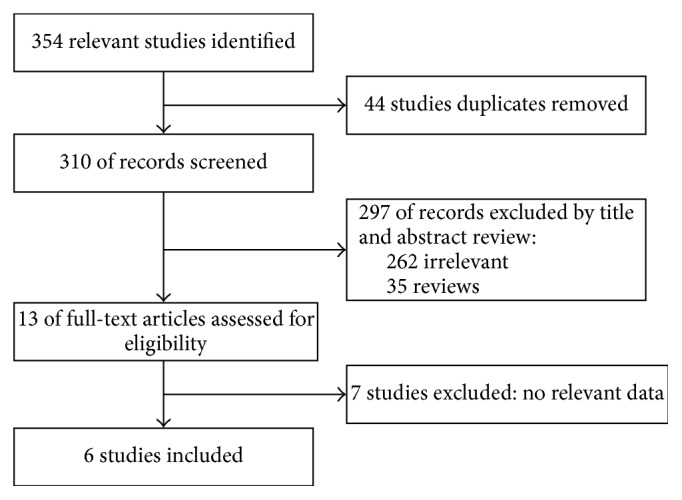
Flow chart of literature search and study selection.

A total of 354 articles were initially reviewed by two independent reviewers to identify the studies that could be included according to the inclusion and exclusion criteria. Among these studies, 44 studies duplicated were excluded. The remaining 304 articles were excluded: 262 irrelevant studies, 35 review articles, and 7 studies not reporting relevant survival data (Figure 1). Six studies with five retrospective cohorts and one randomized controlled trial (RCT) study were included finally [14–19]. The detailed information of the included studies was shown in Table 1.

A total of 994 patients were included in this meta-analysis, among which 409 were in NA-treated group whereas 585 in control group. Four studies applied transarterial chemoembolization (TACE) and two applied radiofrequency ablation (RFA) as unresectable treatment for chronic hepatitis B virus-related HCC. The characteristics and quality of the studies are summarized in Table 1. Nucleotide analogs lamivudine alone was used in the study from Yoshida et al. and Xu et al. [16, 17]. In other studies, lamivudine, adefovir, telbivudine, and entecavir were used. Lamivudine-resistant patients were treated with adefovir and entecavir alone or in combination.

### 3.2. Overall Survival Rate

**Figure 2:**
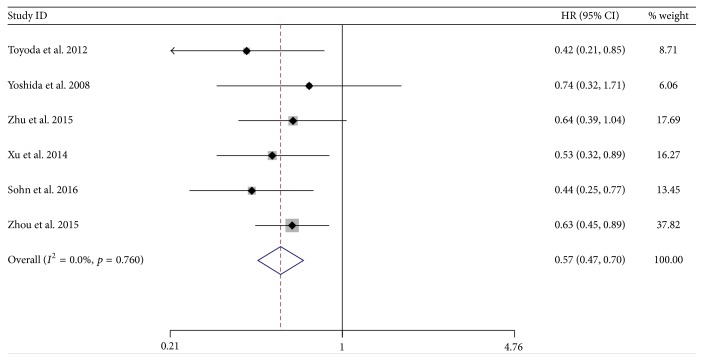
Forrest plot for overall survival after unresectable treatment in patients of HBV-related HCC.

**Figure 3:**
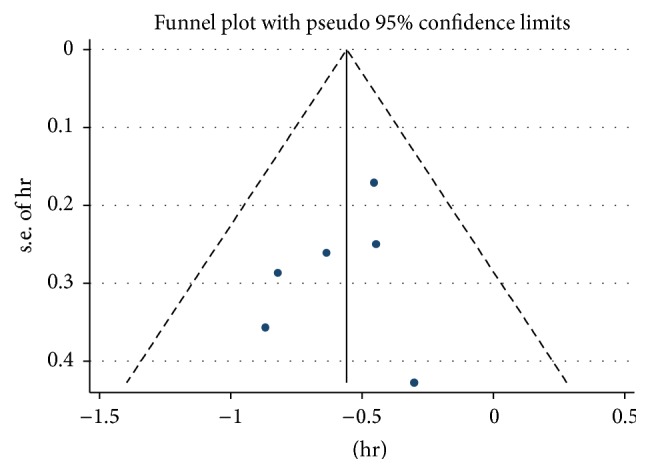
Funnel plot for assessing publication bias.

There was a significant difference between NA-treated and control group by pooling the data from the six studies which reported OS. The results showed a significantly lower hazard of death among the NA-treated group (HR = 0.57; 95% CI = 0.47–0.70;* p* < 0.001) with low heterogeneity (*χ*^2^ = 2.61; degrees of freedom [d.f.] = 5;* p* = 0.760;* I*^2^ = 0.0%) (Figure 2). No significant publication bias was detected by funnel plots (Figure 3).

**Figure 4:**
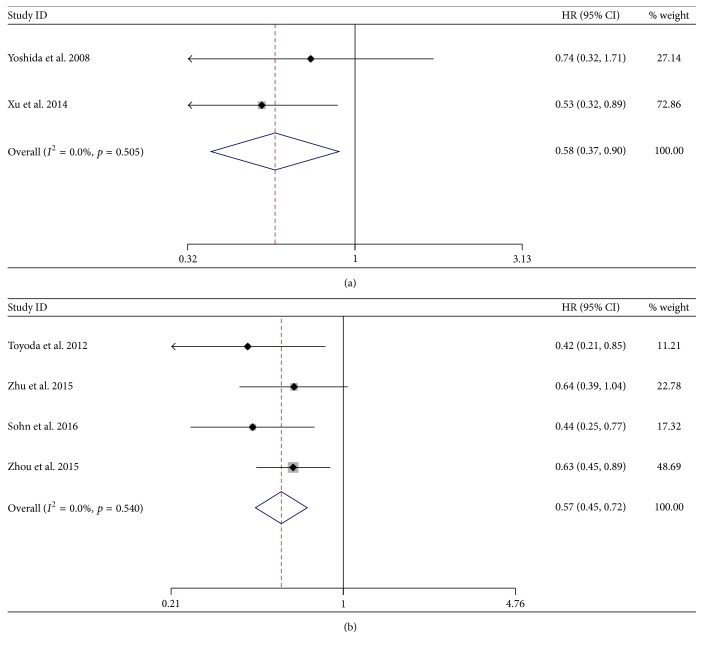
Forrest plot for overall survival after unresectable treatment in patients of HBV-related HCC: (a) patients treated with lamivudine; (b) patients treated with various nucleot(s)ide analogs.

The HRs obtained in the subgroup analysis for patients treated with lamivudine only (HR = 0.58; 95% CI = 0.37–0.90;* p* = 0.015) and patients treated with various NAs (HR = 0.57; 95% CI = 0.45–0.72;* p* < 0.001) were similar. There was no obvious between-study heterogeneity for the LAM subgroup (*χ*^2^ = 0.44; d.f. = 1;* p* = 0.505;* I*^2^ = 0.0%) (Figure 4(a)). Heterogeneity in the various kinds of nucleot(s)ide analogs subgroup was a little large (*χ*^2^ = 2.20; d.f. = 3;* p* = 0.540;* I*^2^ = 0.0%), so we used the random-effect model in analysis (Figure 4(b)).

**Figure 5:**
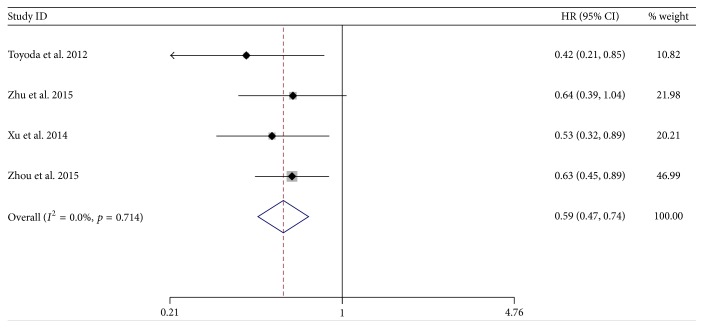
Forrest plot for overall survival after TACE in patients of HBV-related HCC.

In the four studies, in the subgroup of patients treated with TACE, there was a significantly lower hazard of death among the NA-treated group (HR = 0.59; 95% CI = 0.47–0.74;* p* < 0.001) and lower heterogeneity (*χ*^2^ = 1.40; d.f. = 3;* p* = 0.714;* I*^2^ = 0.0%) compared to the control group (Figure 5).

**Figure 6:**
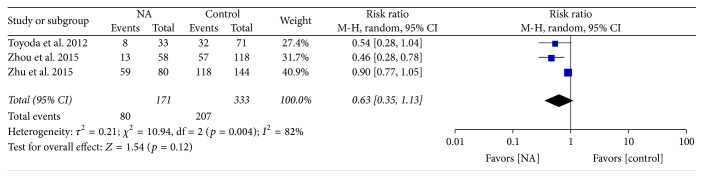
Forrest plot for overall mortality after unresectable treatment in patients of HBV-related HCC.

By pooling the data from three of the six studies which reported overall mortality, the overall mortality was lower in the antiviral group than the control group; however, there was no statistical significance. Among NA patients, the mortality rate was lower by 57% compared to the control patients (RR = 0.43; 95% CI = 0.35–1.31;* p* = 0.12) (Figure 6). The heterogeneity between studies was so large that we used random-effect model.

**Figure 7:**
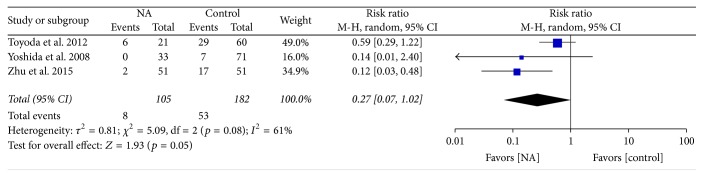
Forrest plot for overall mortality secondary to liver failure after unresectable treatment in patients of HBV-related HCC.

We analyzed the impact of nucleot(s)ide analogs on the mortality secondary to liver failure and showed that 61 patients died of liver failure in three included studies in a total of 287 patients. Among NA patients, the mortality rate was lower by 73% compared to the control patients (OR = 0.27; 95% CI = 0.07–1.02;* p* < 0.05) (Figure 7).

### 3.3. Progression-Free Survival Rate

**Figure 8:**
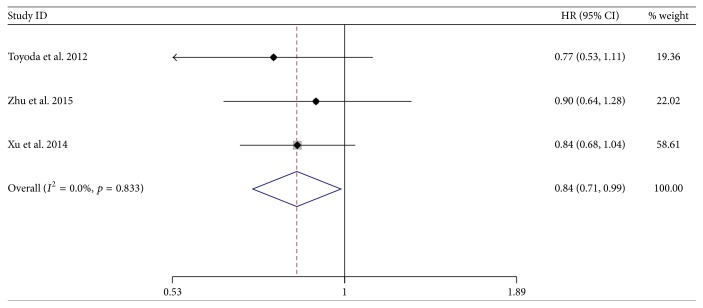
Forrest plot for progression-free survival after unresectable treatment in patients of HBV-related HCC.

There was also a significant difference between two groups by pooling the data from three of the six studies which reported PFS. The result favored NA-treated group (HR = 0.84; 95% CI = 0.71–0.99;* p* = 0.034) with no heterogeneity (*χ*^2^ = 0.36; degrees of freedom [d.f.] = 2;* p* = 0.833;* I*^2^ = 0.0%) (Figure 8).

### 3.4. Sensitivity Analysis

**Figure 9:**
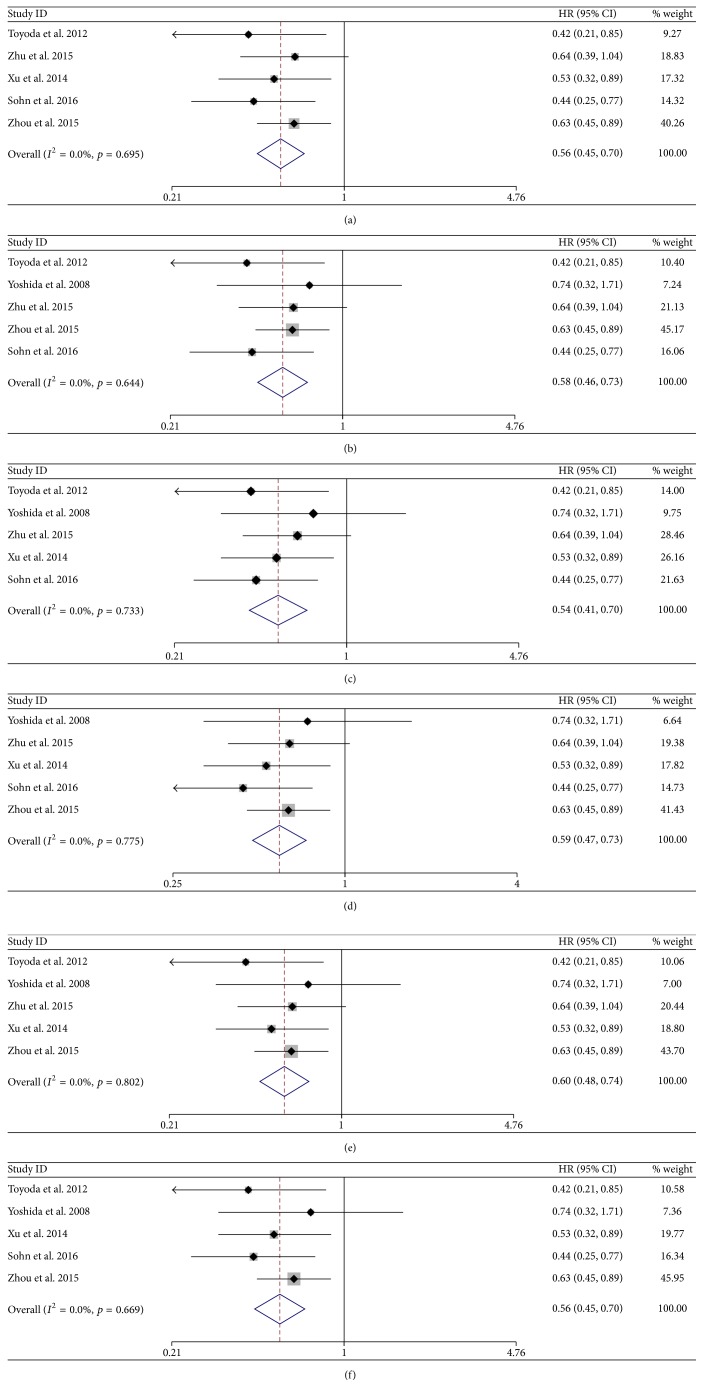
Sensitivity analyses for overall survival after unresectable treatment in excluding each study one by one. Sensitivity analysis for overall survival after unresectable treatment in excluding study from Yoshida et al. 2008 (a), Xu et al. 2014 (b), Zhou et al. 2015 (c), Toyoda et al. 2012 (d), Sohn et al. 2016 (e), and Zhu et al. 2015 (f).

To evaluate the stability of the results, a sensitivity analysis was performed in the subgroup of five of the six studies after excluding each study one by one in random order. First, we removed the study with maximum HR value (Yoshida et al. 2008); there was a significant difference favoring the NA-treated group versus the control group (HR = 0.56; 95% CI = 0.45–0.70;* p* < 0.001) with low between-study heterogeneity (*χ*^2^ = 2.54; d.f. = 4;* p* = 0.695;* I*^2^ = 0.0%) (Figure 9(a)). Additionally, after removing the RCT study (Xu et al. 2014), there was a significant difference favoring the NA-treated group versus the control group (HR = 0.58; 95% CI = 0.46–0.73;* p* < 0.001) with low between-study heterogeneity (*χ*^2^ = 2.64; d.f. = 4;* p* = 0.644;* I*^2^ = 0.0%) (Figure 9(b)). Other studies were also excluded one by one and performed to further confirm the validity of the results (Figures 9(c)–9(f) and Table 2). All above results indicated that the overall result was not substantially influenced by any single study. The result indicates that the current meta-analyses were comparatively reliable.

## 4. Discussion

Hepatitis virus infection is involved in the development of hepatocellular carcinoma. Recent studies have shown that antiviral therapy is associated with high survival of patients with HBV-related HCC after curative treatment [5, 6]. However, survival rate in patients with unresectable HBV-related HCC after treatment with nucleot(s)ide analogs is not definite. The results of this meta-analysis showed that nucleot(s)ide analogs therapy could improve overall survival (HR = 0.57) compared to control group without antiviral therapy in HBV-related HCC. Concerning the outcome of overall mortality, NA-treated group exerted a lower death rate (37%) relative to the control group; however, there was no statistical significant difference between the two groups. The results from previous studies have shown that antiviral therapy with nucleot(s)ide analogs is beneficial in improving liver function and reducing the incidence of HCC in patients with HBV [20, 21]. Thus it could be speculated that antiviral therapy would alleviate liver injury, thus reducing the progression of HCC in consideration of less incidence of liver failure. The second outcome of our study was progression-free survival. The result indicated that patients receiving NAs had a lower risk of disease progression or death compared to the control patients (HR = 0.84). In the subgroup of patients that died of liver failure, mortality was also reduced by 73% in NA-treated group, but there was no statistically significant difference. We attempted to perform a subgroup analysis on the type of liver function. However, we cannot perform the analysis since the required data cannot be extracted.

Nucleot(s)ide analogs including lamivudine (LAM), adefovir (ADV), telbivudine (LDT), clevudine (CLV), entecavir (ETV), and tenofovir (TDF) were searched to fulfill the criteria that could be included in the meta-analysis. However, it has not been reported whether tenofovir treatment is better than placebo or no antiviral therapy in unresectable HBV-HCC. Drug resistance was the main concern in long-term antiviral therapy.

It was reported that patients treated by LAM exerted the greatest drug resistance relative to those treated by other nucleot(s)ide analogs [22]. In addition, we performed subgroup analysis. We showed that the HR values of the LAM-treated group were similar to the HR values of others NA-treated group. The HR values in the two subgroups were 0.58 and 0.57, respectively.

TACE is one of the palliative treatments of HCC. In the included studies, four studies applied TACE while two studies applied RFA. We performed a subgroup analysis of patients treated with TACE. The result of the subgroup (HR = 0.59) was nearly identical to the primary outcome of all six included studies (HR = 0.57). The result confirmed the effect of antiviral therapy since the way of unresectable treatment had little influence on the HR between two groups.

Sensitivity analysis was performed to confirm the robustness of the results. Six studies including five retrospective cohort studies and one RCT study fulfilled our criteria. By removing one RCT study, we pooled the data from the remaining five retrospective cohort studies. We found that the HRs of the sensitivity analysis (HR = 0.58) and overall meta-analysis (HR = 0.57) were nearly the same. It suggested that our results were reliable.

There was also a limitation in this meta-analysis. There were only six studies fulfilling the criteria and five of these studies were retrospective cohort studies; hence this study had no sufficient data from RCTs. However, antiviral therapy was suggested to be used in patients with HBV in most guidelines and it seemed that performing a RCT was unethical.

In summary, despite the limitation reported above, we still concluded that nucleot(s)ide analogs are beneficial in improving the survival rate after unresectable treatment in patients of HBV-related HCC.

## Figures and Tables

**Table 1:** Characteristics of the included studies.

Study	Nature of study	Number of patients (T/C)	Male/female	Age (T/C)	Unresectable treatment	Antiviral therapy	Follow-up (months)	Outcomes
Toyoda et al. 2012	Retrospective cohort	81 (21/60)	67/14	60.3/60.6	TACE	LAM, ETV, ADV	19.3	OS, PFS
Yoshida et al. 2008	Retrospective cohort	104 (33/71)	78/26	57/59	RFA	LAM	NA	OS, RFS
Zhu et al. 2015	Retrospective cohort	176 (58/118)	152/24	48.7/49.8	TACE	LAM, ADV	NA	OS, PFS
Xu et al. 2014	RCT	181 (92/89)	164/17	56.0/55.1	TACE	LAM	24	OS, PFS
Sohn et al. 2016	Retrospective cohort	228 (125/103)	170/58	55.0/55.2	RFA	LAM, ETV, CLV, ADV, TDF	96	OS
Zhou et al. 2015	Retrospective cohort	224 (80/144)	209/15	48.0/50.5	TACE	LAM, ADV, ETV	9.9	OS

RCT: randomized controlled trials; TACE: transarterial chemoembolization; RFA: radiofrequency ablation; OS: overall survival; PFS: progression-free survival; RFS: recurrence-free survival rate.

**Table 2:** Results of sensitivity analysis.

Study excluded	HR (95% CI)	*I* ^2^	*p*
Yoshida et al. 2008	0.56 (0.45–0.70)	0.0%	0.695
Zhou et al. 2015	0.54 (0.41–0.70)	0.0%	0.733
Sohn et al. 2016	0.60 (0.48–0.74)	0.0%	0.802
Xu et al. 2014	0.58 (0.46–0.73)	0.0%	0.644
Zhu et al. 2015	0.56 (0.45–0.70)	0.0%	0.669
Toyoda et al. 2012	0.59 (0.47–0.73)	0.0%	0.775

HR: hazard ratio.

## Additional Points

*Core Tip*. There is no doubt that nucleot(s)ide analogs (NA) therapy is of benefit to improve survival rate after curative treatment of HBV-related HCC for it has been proved by numerous meta-analyses. However, meta-analysis on benefit of NA therapy in patients after unresectable treatment has never been published before. In this meta-analysis, we draw a conclusion that nucleot(s)ide analogs therapy could improve overall survival significantly. There were also subgroups and sensitivity analyses to make the result more reliable.

## Data Access

No additional data are available.
